# Could a multidisciplinary regional audit identify avoidable factors and delays that contribute to stillbirths? A retrospective cohort study

**DOI:** 10.1186/s12884-020-03402-z

**Published:** 2020-11-16

**Authors:** I. Sterpu, J. Bolk, S. Perers Öberg, I. Hulthén Varli, E. Wiberg Itzel

**Affiliations:** 1 Department of Clinical Science and Education, Karolinska Institutet, Södersjukhuset, Stockholm, Sweden; 2 Department of Obstetrics and Gynecology, Södersjukhuset, Stockholm, Sweden; 3 Clinical Epidemiology Division, Department of Medicine Solna, Karolinska Institutet, Stockholm, Sweden; 4 Sachs’ Children and Youth Hospital, Södersjukhuset, Stockholm, Sweden; 5 Department of Women’s and Children’s Health, Karolinska Institutet, Stockholm, Sweden; 6 Department of Obstetrics and Gynecology, Karolinska University Hospital, Stockholm, Sweden

**Keywords:** Stillbirth, Audit, Quality of care, Causes of death, Preventable stillbirth

## Abstract

**Background:**

The annual rate of stillbirth in Sweden has remained largely unchanged for the past 30 years. In Sweden, there is no national audit system for stillbirths. The aim of the study was to determine if a regional multidisciplinary audit could help in identifying avoidable factors and delays associated with stillbirths.

**Methods:**

Population-based retrospective cohort study.

*Settings*: Six labour wards in Stockholm County.

*Participants*: Women delivering a stillbirth > 22 weeks of gestation in Stockholm during 2017.

***I****ntervention*: A multidisciplinary team was convened. Each team member independently assessed the medical chart of each case of stillbirth regarding causes and preventability, level of delay, the standard of healthcare provided, the investigation of maternal/foetal diseases and if any recommendations were given for the next pregnancy. A decision was based on the agreement of all five members. If no agreement was reached, a reassessment of the case was done and the medical record was scrutinized again until a mutual decision was made.

*Primary outcomes*: The frequency of probably/possibly preventable factors associated with a stillbirth and the level of delay (patient/caregiver).

*Secondary outcomes*: The causes of death, the standard of antenatal/intrapartum/postpartum care, whether a summary of possible causes of the stillbirth was made and if any plans for future pregnancies were noted.

**Results:**

Thirty percent of the stillbirths were assessed as probably/possibly preventable. More frequent ultrasound/clinical check-ups, earlier induction of labour and earlier interventions in line with current guidelines were identified as possibly preventable factors. A possibly preventable stillbirth was more common among non-Swedish-speaking women (*p* = 0.03). In 15% of the cases, a delay by the healthcare system was identified. Having multiple caregivers, absence of continuity in terms of attending the antenatal clinic and not following the basic monitoring program for antenatal care were also identified as risk factors for a delay.

**Conclusion:**

A national/regional multidisciplinary audit group retrospectively identified factors associated with stillbirth. Access to good translation services or a more innovative approach to the problem regarding communication with mothers could be an important factor to decrease possible patient delays contributing to stillbirths.

**Trial registration:**

NCT04281368.

## Background

Each year, 2.6 million stillbirths occur worldwide [1]. The use of different clinical definitions of stillbirth, the abundance of cause of death classification systems and the variation in record keeping could, at least partially, explain why the rate of stillbirth varies between countries with similar income levels and levels of medical care. According to a ranking made by World Health Organization (WHO) 2009, out of 194 countries Sweden had the 12th lowest stillbirth rate.

Sweden changed the definition of stillbirth (from foetal death from gestational week 28 + 0 to foetal death from gestational week 22 + 0) in June 2008 in agreement with the recommendation of the International Classification of Diseases (ICD-10) and WHO [2, 3]. Since then, the yearly incidence has varied between 3.7 out of 1000 births and 4.1 out of 1000 births. This incidence was higher than deaths from any other single disease in Sweden for all ages, deaths related to infections (0.21 out of 1000), deaths related to cancer (2.37 out of 1000) and deaths related to cardiovascular diseases (3 out of 1000) in 2017 [4].

Despite multiple information campaigns directed towards pregnant women and healthcare workers by the Swedish National Board of Health and Welfare and a high proportion of pregnant women attending antenatal care, the annual rate of stillbirth in Sweden has remained largely unchanged for the past 30 years. In contrast, using standardized information [5] about reduced foetal movements, it was found that in Norway there was a reduction in the incidence of stillbirths from 4.2‰ to 2.4 ‰ in a group with reduced foetal movements and from 3‰ to 2‰ in the general population.

Stillbirth is a traumatic experience that has a significant impact on the family. It is associated with a higher risk of depression, anxiety, post-traumatic stress disorder and the parents’ separation compared to live births. It also has an impact on the mental and physical health of the siblings [6–8]. At the same time, stillbirths have a profound effect on the medical staff attending the parents [9, 10] and a negative economic impact on society [11, 12].

During 2011–2016, a series of articles published in *The Lancet* underlined an urgent need for action regarding stillbirths and the importance of a more standardized way of data collection, investigation and classification [1, 12–16]. Using audits has been suggested as a way to map the circumstances surrounding a stillbirth. Previous studies suggest that the implementation of regional audits can improve the registration of stillbirths and causes of death, help identify the cases with suboptimal care and possibly help decrease the number of preventable stillbirths [17, 18], even though more evidence for this in high-income settings is needed [19].

Comparison between different countries has been difficult due to different classification systems. However, three of the high-income countries (UK, New Zealand and the Netherlands) that use an active structured national audit system for stillbirths have reported a gradual decline in the rate of stillbirth [20].

In Sweden, there is no national audit system for stillbirths and, to our knowledge, no previous reports of any structured regional population-based audits of stillbirths focusing on possibly/probably preventable factors and delays that might have contributed to the stillbirths. Our aim with this project was to explore whether using a regional population-based multidisciplinary audit could help in identifying modifiable factors and delays that could contribute to a stillbirth. A secondary objective was to assess whether the Swedish national recommendations for a clinical protocol to investigate stillbirths was followed and whether there was a case summary with recommendations for future pregnancies.

## Methods

### Study design

This is a retrospective cohort study based on medical chart reviews and audit meetings.

### Study population

Inclusion criteria: All stillbirths in the Stockholm region during 2017. The stillbirths were identified from the obstetric record system Obstetrix (Cerner Sverige AB), which is used in all delivery wards and antenatal care (ANC) units in Stockholm. Stillbirth was defined based on the ICD-10 definition as foetal death at ≥22 + 0 weeks of gestation.

Exclusion criteria: Registered stillbirths where the audit group assessed that the normal evolution of the pregnancy had stopped before 22 + 0 weeks of gestation.

### Data collection

Data regarding the maternal background, the pregnancy characteristics and the diagnostic investigations of the stillbirth were collected from the obstetric record system Obstetrix and TakeCare (TakeCare CompuGroup Medical Stockholm AB). Medical records were assessed from the first registration of the pregnancy at the ANC clinic until the last check-up 6–12 weeks postpartum. Data about all deliveries in Stockholm and all stillbirths in Sweden in 2017 were collected from the Swedish Medical Birth Register with the help of the Swedish National Board of Health and Welfare. The register gathers medical information from all maternal healthcare givers and deliveries in Sweden with almost complete coverage (missing data of 1–4%) [21].

Small for gestational age (SGA) was defined in conformity with the national guidelines as a birth weight deviation ≤22%, that is, below two standard deviations from the expected average weight for gestational age [22]. The gestational age was based on a routine ultrasound examination that all women in Sweden are offered free of charge as part of routine ANC in the first or second trimester. Routine ultrasound attendance is around 98% [23], and 98.7% of pregnant women have at least one ANC visit during pregnancy. The country of birth was retrieved from the ANC medical records. The participants were divided into two groups, Swedish-speaking and non-Swedish-speaking, depending on the necessity for a professional translator when dealing with medical staff. All information about the cases was recorded in a pseudonymized database from which the unique personal identifier number assigned to all Swedish citizens at birth or immigration was removed and replaced by a serial number [24].

Any information on reduced foetal movements was collected from the medical charts. Current management of reduced foetal movements in Stockholm is based on the recommendations of the Swedish National Board of Health and Welfare [25] and consists of cardiotocography (CTG) assessment for 20 min with an additional ultrasound to assess foetal movements and amniotic fluid if the decreased foetal movements persist during the visit to the obstetrician/midwife.

### Audit method

A multidisciplinary team consisting of three obstetricians (IS consultant and EWI, IHW senior consultants), one midwife (senior midwife with many years of clinical experience) and one neonatologist (JB senior consultant) was assembled. The team represented the two largest hospitals in the region, Karolinska University Hospital and Soder Hospital, both in Stockholm. Each team member in the audit group had access to all the medical patient files from the hospitals and the majority of the files from the ANC clinics. Each team member independently assessed each case regarding causes of death, probably/possibly preventable/non-preventable deaths, from the perspective of the healthcare system. Further, the audit members assessed the level of delay, if any; the standard of healthcare provided to the patients with stillbirth; the post-mortem examinations of infants; investigations of maternal/foetal diseases of importance; and if there were any recommendations given for the next pregnancy. The audit group assembled on several occasions for discussion and the individual assessment of each case. To enable an ethical discussion, every member of the audit group signed a confidentiality form, and all cases were assessed by comparison with local guidelines in praxis.

### Outcomes

The primary outcomes were probably/possibly preventable/non-preventable deaths and the level of delay (patient or caregiver) if any. The secondary outcomes were causes of death; standard of antenatal, intrapartum and postpartum care; whether a summary of the possible cause of death was made; and whether there was a clinical plan for the next pregnancy.

Preventable stillbirth was classified as death that might have been avoided/prevented with better healthcare, better guidelines or better patient compliance. The classification was done in two steps after the audit members had done their individual assessment. First, the audit group decided on the cause of death and if there were any cases involving what was considered substandard care, incorrect management or poor patient compliance. Second, the audit group decided on the preventability of the deaths. The categorization was determined by answering the question ‘Was the death preventable by better quality of care/routines or patient compliance?’ and by using a simplified Likert scale (probably preventable, possibly preventable and probably not preventable). Each case was assessed individually by each member of the audit group and then discussed in meetings. A decision was based on the agreement of all five members. If no agreement was reached, a reassessment of the case was done on the spot and the medical record was scrutinized again until a mutual decision was made.

The level of delay was classified as healthcare- or patient-associated and defined as shown below, a simplified version of the avoidable factors associated with perinatal death from the perinatal problem identification program [26].

### The level of delay

**Table:** 

**Patient delay**	Did not initiate antenatal care
	Infrequent visits to antenatal clinic/maternity
	Delayed seeking medical care in labour, reduced foetal movements or other complications of pregnancy
	Declined admission/treatment
	Alcohol/drug abuse
	Failed to return on prescribed date
**Healthcare delay**	No advice or inadequate advice given to the mother
	Delay in taking in the patient
	Delay in referring the patient for secondary/tertiary treatment
	Foetal distress not detected, intrapartum foetus monitored/not monitored
	Incorrect medical management

The causes of death were categorized in accordance with the Stockholm classification of stillbirths, which allows for more than one cause of stillbirth and consists of 17 groups where most groups are subdivided into definite, probable and possible relationship to stillbirth. The subdivision is not based on strict criteria, but, as is common in clinical work, on partly subjective assessments. The subdivision was a way to include etiological factors and diseases that could contribute to foetal death and thereby allow the classification to include causes of death that individually are too weak or too rare to result in a definite association with cause of death [27].

Substandard care was defined as non-compliance to guidelines or missing local guidelines and/or deviations from standard professional care. The quality of care was analysed in terms of ANC and intrapartum and postpartum investigation of the stillbirth.

The standard investigation protocol after the occurrence of a stillbirth in Stockholm is presented below. There are small differences regarding the protocol of investigations between the six labour wards in Stockholm. Some protocols also include a bacterial urine culture and the extended coagulation profile as standard elements.

### Investigation protocol for stillbirths

**Table:** 

**Sample**	**Analysis**
**Maternal**	
Blood sample	Hemoglobin
	EVF
	Foetal haemoglobin
	Antithrombin
	PK/INR
	Activated partial thromboplastin time
	Alanine aminotransferase (ALT)
	Serum bile acids
	Lupus anticoagulant (LA)
	Anticardiolipin antibody (aCL)
	Factor V Leiden mutation
	Serology; Toxoplasma, Rubella, Herpes simplex, cytomegalovirus and Parvovirus B19
Cervix	Bacterial culture
**Foetal**	
Foetal heart blood	Bacterial culture
Placenta	Karyotype Pathologic-anatomic examination
Foetus	Autopsy

### Statistical analysis

Data were analysed using SPSS software version 23.0 (IBM Corp, Armonk, NY, USA). Categorical variables are presented in frequencies and continuous variables are presented as mean value with standard deviation. Histograms were initially used to assess data distribution. As there were no differences in significance between the mean and the median, all continuous variables are presented as means with SD. To test group differences, we used an independent t-test for the continuous variables and relative risk with 95% confidence interval (CI) or a Fishers exact test for the categorical variables. The level of significance was set at a *p* value less than 0.05 for the continuous variables and a relative risk not crossing one for the categorical variables.

### Patient and public involvement

No patients were involved in the design, analysis or report of the study. The public was represented by the Swedish National Board of Health and Welfare.

## Results

In 2017, Stockholm had a population of approximately 2.3 million, with a total of 28,805 deliveries distributed among the six delivery clinics. In 2017, there were 82 registered stillbirths in Stockholm; three were categorized by the audit group as missed abortions and were therefore excluded from the study. The remaining 79 stillbirths (78 women) were analysed by the audit group.

The demographic details, pregnancy characteristics and neonatal outcomes of the women with stillbirths and live births in Stockholm are presented in Table 1. The stillbirth group had a higher rate of SGA (40.5%) compared to the live birth group (RR 10.3, 95% CI 7.88–13.46). Women born in Africa were overrepresented in the stillbirth group (19.2%) compared to the live birth group (RR 2.43 (1.53–3.87).

**Table 1:** Baseline data for group with stillbirths (78 women, 79 babies) and the group with live births (*n* = 28,584)

	Stillbirths ***n*** = 78 mean (SD) or n (%)	Live births ***n*** = 28,584 mean (SD) or n (%)	***p*** value^**a**^/ Relative risk (95% CI)
**Maternal characteristics**			
Age (years)	32.2 (5.1)	32.4 (5.2)	0.73
BMI^b^	25.5 (4.4)	24.4 (4.4)	0.03
Smokers^c^	4 (5)	849 (3)	1.68 (0.65–4.39)
History of psychiatric comorbidity	9 (11.3)	3579 (13)	0.91 (0.49–1.68)
In vitro fertilization	4 (5)	1827 (6.5)	0.78 (0.29–2.02)
Nullipara	20 (25.6)	12,257 (43.5)	0.58 (0.39–0.85)
Duplex	5 (6.4)	877 (3.07)	2.06 (0.88–4.83)
At least one previous miscarriage	26 (33.3)	6858 (23.9)	1.42 (1.05–1.94)
Complications of pregnancy^d^	9 (11.5)	1678 (6.5)	1.75 (0.94–3.24)
**Socio-economic situation**			
Living with the father of the child	69 (87.3)	25,927 (93.7)	0.84 (0.75–0.94)
Single mother	4 (5)	539 (1.95)	2.59 (0.99–6.77)
Education: more than 3 years postsecondary	29 (39.7)	13,284 (47.5)	0.77 (0.57–1.03)
Working fulltime	38 (48.7)	16,902 (64.1)	0.58 (0.44–0.75)
Country of birth			
Sweden	33 (42.3)	18,807 (66.8)	0.58 (0.45–0.75)
Asia	13 (20.5)	3973 (14.1)	1.16 (0.74–1.80)
Africa	15 (19.2)	1771 (6.3)	2.43 (1.53–3.87)
**Onset of labour**			
Spontaneous	17 (21.5)	19,560 (68.4)	< 0.01
Induced	53 (67.1)	5337 (18.7)	
Caesarean (ES + AS)	9 (11.4)	3684 (12.9)	
**Method of delivery**			
Spontaneous vaginal	67 (84.8)	20,908 (73.4)	0.34
Vacuum	2 (3)	1586 (5.6)	
Caesarean (ES + AS)	10 (12.6)	6002 (21)	
**Infant characteristics**			
Gestational age (days)	231 (43.5)	277 (13.6)	< 0.01
22–32 weeks of gestation	33 (41.8)	308 (1.8)	39.4 (29.87–52.08)
Term pregnancies	27 (34.2)	25,230 (88.3)	0.38 (0.28–0.51)
Birth weight (g)	1953 (1241)	3480 (584.1)	< 0.01
Length (cm)	42 (10.1)	50 (2.7)	< 0.01
Female gender	41 (51.8)	14,352 (49.4)	1.04 (0.85–1.28)
Malformations	6 (7.6)	1041 (3.64)	2.05 (0.95–4.46)
Small for gestational age	32 (40.5)	1144 (3.9)	10.3 (7.88–13.46)

^a^*p* value significant if < 0.05; the differences between the groups are presented as relative risk with a 95% Confidence interval

^b^BMI = Body Mass Index = weight in kg/height x height in m2, calculated at the beginning of pregnancy

^c^Smokers at the beginning of pregnancy

^d^Gestational diabetes, preeclampsia, hypertension, or cholestasis of pregnancy; the continuous variables are presented as mean and SD, and the categorical variables are presented as numbers (percentages of total)

*ES* elective caesarean section, *AS* acute caesarean section

To determine if the stillbirth group in Stockholm were representative of all stillbirths in Sweden during the same year, we compared the groups considering specific data (see Table 2). We found there were fewer nulliparous women, more complications of pregnancy, more women born in Africa and more infants with SGA in the Stockholm stillbirth group compared to the total Swedish population of stillbirths.

**Table 2:** Stillbirths in Stockholm (78 women, 79 babies) and stillbirths in Sweden (314 women and 336 babies), 2017

	Stillbirths Stockholm *n* = 78 mean (SD) or n (%)	Stillbirths Sweden *n* = 314 mean (SD) or n (%)	***p*** value^**a**^/
**Maternal characteristics**			
Age (years)	32.2 (5.1)	30.4 (5.5)	< 0.001
BMI^b^	25.5 (4.4)	26.6 (5.4)	0.09
Smokers^c^	4 (5)	18 (5.7)	0.80
In vitro fertilization	4 (5)	18 (5.7)	0.80
Nullipara	20 (25.6)	156 (49.7)	< 0.001
Duplex	5 (6.4)	22 (7)	0.47
At least one previous miscarriage	26 (33.3)	71 (22.6)	0.05
Complications of pregnancy^d^	9 (11.5)	3 (0.9)	< 0.001
**Socio-economic situation**			
Living with the father of the child	69 (87.3)	266 (84.7)	0.66
Single mother	4 (5)	7 (2.2)	0.17
Education: more than 3 years postsecondary	29 (39.7)	94 (29.9)	0.09
Working fulltime	38 (48.7)	118 (37.6)	0.09
Country of birth			0.13
Sweden	33 (42.3)	216 (64.3)	
Asia	17 (21.8)	57 (16.9)	
Africa	15 (19.2)	34 (10.1)	
Europe	2 (2.6)	18 (5.4)	
South America	2 (2.6)	1 (0.3)	
Unknown/Missing	5 (6.4)	10 (3)	
**Onset of labor**			0.92
Spontaneous	17 (21.5)	69 (20.5)	
Induced	53 (67.1)	230 (68.5)	
Caesarean (ES + AS)	9 (11.4)	36 (10.7)	
**Method of delivery**			0.43
Spontaneous vaginal	67 (84.8)	287 (85.4)	
Vacuum	2 (2.5)	2 (0.6)	
Caesarean (ES + AS)	10 (12.6)	47 (13.9)	
**Foetal characteristics**	*n* = 79	*n* = 336	
22–32 weeks	33 (41.8)	137 (40.8)	0.65
Term pregnancies	27 (34.2)	121 (36)	0.39
Birth weight (g)	1953 (1241)	2029 (1231)	0.65
Length (cm)	42 (10.1)	43 (9.7)	0.41
Female gender	41 (51.8)	170 (50.6)	0.84
Malformations	6 (7.6)	6 (1.7)	< 0.001
Small for Gestational Age	32 (40.5)	97 (28.9)	0.03

^a^*p* value significant if < 0.05

^b^BMI = Body Mass Index = weight in kg/height x height in m2, calculated at the beginning of pregnancy

^c^Smokers at the beginning of the pregnancy

^d^Gestational diabetes, preeclampsia, hypertension, or cholestasis of pregnancy

The continuous variables are presented as mean and SD

The categorical variables are presented as numbers (percentages of total)

*ES* elective caesarean section, *AS* acute caesarean section

Twenty-four of the 79 stillbirths (30.4%) were assessed as probably/possibly preventable. Twenty-nine of the 35 early stillbirths (82.9%) and 26 of the 44 late stillbirths (59.1%) were described as not preventable (*p* = 0.12). There was no statistically significant difference between the groups regarding probably preventable stillbirths when divided by country of the mother’s birth (not Sweden/Sweden): 7 out of 40 (17%) vs. 5 out of 34 (14%) (*p* = 0.36). However, there was a statistically significant difference regarding Swedish-speaking/non-Swedish-speaking women when comparing possibly preventable, probably preventable and non-preventable stillbirth (*p* = 0.03, Table 3).

**Table 3:** Main results for preventable stillbirths, any delay and any substandard care in Stockholm, 2017 and analysis for the subgroups of Swedish-speaking/non-Swedish-speaking women

	Count (*n* = 79)	Percent (%)	Swedish-speaking *n* (%)	Non-Swedish-speaking n (%)	*p* value^a^
**Preventable**					0.03
Probably yes	13	17	9 (14)	4 (25)	
Possibly yes	11	14	6 (10)	5 (31)	
Probably no	55	70	48 (76)	7 (44)	
**Delay**					0.02
No delay	54	68	47 (75)	7 (44)	
Patient	13	17	7 (11)	6 (37)	
Healthcare	12	15	9 (14)	3 (19)	
**Substandard care antenatal**					0.47
	22	28	17 (27)	5 (31)	
**Substandard care postnatal**					
Incomplete protocol	14	18	13 (21)	1 (6)	0.28
Autopsy (yes)	52	66	44 (70)	8 (50)	
Placenta exam (yes)	79	100	63 (100)	16 (100)	
Karyotype (yes)	71	90	55 (87)	16 (100)	

^a^*p* value significant if < 0.05

Approximately 70% of cases of stillbirth in this project were assessed as not preventable. In some of these cases, there was an explanation for the death, such as known malformations of the foetus, known chromosomal abnormalities or extreme prematurity. In the remaining 30%, we identified factors such as more frequent ultrasound/clinical check-ups, earlier induction of labour and earlier interventions in line with current guidelines that could probably/possibly prevent the stillbirths. The committee agreed in all cases except two, where a decision was reached by majority vote. We identified having multiple caregivers (seeking medical help in different hospitals), absence of continuity in the ANC and not following the basic monitoring program for pregnancy as additional risk factors for stillbirth.

In 54 of the 79 stillbirths (68.4%), the audit group assessed that there was no patient or healthcare provider delay. A delay by the healthcare system could be identified in 12 of the 79 cases (15%) (Table 3). In the group of non-Swedish-speaking women, there was a higher percentage of patient-related delay compared with the Swedish-speaking group (37% vs. 11%, *p* = 0.02).

Seventy-four of the 79 stillbirths (93.7%) were antepartum and five were intrapartum. The assessed leading cause of stillbirth was intrauterine growth retardation (IUGR)/placental insufficiency, followed by infection (Table 4). Six out of the 79 cases (7.6%) had an unknown/unexplained cause of death.

**Table 4:** Main diagnoses of stillbirths in Stockholm, 2017 (*n* = 79)

Causes of stillbirth	n	%
Malformation and chromosomal abnormalities	6	7.6
Infection	12	15.2
Immunization	0	0
Feto-maternal transfusion	1	1.3
Twin to twin transfusion syndrome	3	3.8
Birth hypoxia	0	0
IUGR/placental insufficiency	31	39.2
Umbilical cord complications	3	3.8
Placental abruption	3	3.8
Preeclampsia	5	6.3
Diabetes mellitus	3	3.8
Intrahepatic cholestasis of pregnancy	2	2.5
Uterine complications	1	1.3
Coagulation disorders	0	0
Other causes	3	3.8
Unknown/Unexplained	6	7.6

*IUGR* Intrauterine Growth Restriction

Forty-eight of the 79 cases (60.8%) had more than one diagnosis that was associated with the stillbirth. The most frequent diagnosis was IUGR/placental insufficiency. This occurred in 10 of the 48 cases (20.8%), followed by infection in six of the 48 cases (12.5%).

The level of strength of the association between the main diagnosis and the stillbirth was categorized into four groups: a definitive association between the diagnosis and the stillbirth in 44 of the 79 cases (55.7%), a probable association in eight of the 79 cases (10.1%), a possible association in 21 of the 79 cases (26.6%) and an unknown association in six of the 79 cases (7.6%).

The audit group assessed that substandard ANC occurred in 22 of the 79 cases (28%). Elements of substandard care, such as incomplete diagnostic protocol for stillbirth, were registered in 14 of the 79 cases (17.7%) (Table 3). The blood sample from the foetal heart was missing in 27 of the cases (34%), in some of these cases due to technical sampling difficulties (the heart blood was sampled but was insufficient to analyse). Furthermore, in 16 of the cases (20.3%) an assessment of the case and cause of stillbirth was not documented in the medical records.

## Discussion

To our knowledge, this is the first report of a multidisciplinary audit of stillbirths in Sweden with a focus on possibly preventable stillbirths, delays and substandard care. The audit was carried out on behalf of the Swedish National Board of Health and Welfare and was performed by a multidisciplinary team consisting of three obstetricians, one midwife and one neonatologist. A combination of different protocols used internationally in similar projects was used [17, 18, 26, 28]. The WHO’s “Making every baby count: audit and review of stillbirths and neonatal deaths” suggests that the audit of medical records may be a tool when trying to identify modifiable factors or avoidable patterns [29]. This was one reason why this audit of all stillbirths in Stockholm was performed.

### Comparing stillbirths in Stockholm with stillbirths in Sweden

The study population in this audit was overall representative of all the stillbirths in Sweden occurring in the same year, except that there were fewer nulliparous women, more complications of pregnancy, more women born in Africa and more infants with SGA in the Stockholm group. The difference was statistically significant and might describe a true difference between the stillborn group in Stockholm and the rest of the country. However, as the study population in Stockholm was small it cannot be ruled out that there is some bias in medical care or that socioeconomic circumstances or simply chance can explain this. The information was also collected in different ways since the Stockholm data came directly from studying the individual medical charts, whereas the data from the whole of Sweden came from national registers.

### Comparing stillbirths with live births in Stockholm

The stillbirths in our population were more often SGA compared to liveborn infants. This is in line with previous reports [30] and is a well-established association in the clinical setting. There was an overrepresentation of women born in Africa in the stillbirth group. This is also in line with previous reports, both in a Swedish register-based study on stillbirths and in an Italian audit on stillbirths [17, 31]. It is also possible, even if we could not control for it in this study, that women from Africa are in a more socioeconomic vulnerable situation. However, adjusting for socioeconomic status did not decrease the overrepresentation of mothers born in Africa in a large register-based study of stillbirths in Sweden, [31] therefore increasing the risk of delay. Our study also indicates that socioeconomic vulnerabilities, such as fewer women working fulltime, could be more common in the stillbirth group.

### Preventable/non-preventable stillbirths

In our cohort, more than two thirds of the stillbirths were assessed as not preventable, and it was more common among stillbirths in early pregnancy. Even though the birth country of the mother did not differ between mothers with preventable and non-preventable stillbirths, it was more common for mothers to be non-Swedish-speaking in the stillbirths that were categorized as probably/possibly preventable. In the Swedish register-based study mentioned above, the authors found that mothers who had been in Sweden less than 5 years had the most increased risk of stillbirth [31]. This is in line with our finding that women with possibly/probably preventable stillbirths were more often non-Swedish-speaking.

In almost one third of the cases of stillbirth in this audit, there was an observed delay of care. When investigating patient-related delay, we found there was a higher rate of patient-related delay in women who were non-Swedish-speaking. Ethnicity and lack of language skills have previously been reported to contribute significantly to stillbirth [32–34]. What seems relevant in our study is whether the woman speaks Swedish or not. Communication problems due to language barriers could be a limiting factor in the situation of threatening stillbirth, partly due to the difficulty of seeking contact with healthcare (as almost all contact in Swedish maternal care and healthcare in general is preceded by telephone contact) and partly due to difficulties in understanding the healthcare system. It is possible that it is more difficult for non-Swedish-speaking women to find information, to describe problems over the telephone when needing healthcare and to understand the information given by healthcare workers over the phone. As midwives pointed out in a survey by the National Board of Health and Welfare, this is often a limiting factor in their work as midwives.

Using interpreters might reduce misunderstandings in the healthcare of non-Swedish-speaking pregnant women, and allowing a non-relative, Swedish-speaking woman from the same cultural background to support and help the pregnant woman can be essential. Previous studies have shown that the assistance of a doula during pregnancy results in better obstetrical and neonatal outcomes [35–37]. The contact between the pregnant woman and the healthcare system might be facilitated by allowing a non-relative, Swedish-speaking woman from the same cultural background, a ‘culture doula’, to support and help the pregnant woman. We therefore suggest that the usefulness of ‘culture doulas’ be further investigated.

### Causes of death

Most of the stillbirths in this audit occurred antepartum (94%). In other similar projects, such as ‘Each baby counts’ in the United Kingdom [28], more cases of intrapartum deaths were sometimes included in the reports. This means that different numbers are presented and that the results could be hard to compare. Furthermore, the mechanisms for the antepartum and intrapartum stillbirths are different and while the former are more likely to depend on the antenatal care organization, the latter are more likely to depend on the quality of labour ward.

When examining if an explanation for the stillbirth could be found in the medical charts, a definite/probable or possible cause of death was found in all but six cases (7.6%). This is low compared to some other reports on stillbirth; however, there are around 35 classification systems for stillbirths, and some of the more complex ones classify approximately 20–30% of cases as unexplained [38, 39]. Despite extensive investigations, no real cause of death could be identified in these cases. The cases where no explanation for the stillbirth can be found are perhaps the most difficult cases for the affected families. A sensible explanation of what has happened can be a consolation in the grief process [40]. According to our clinical experience, not getting an explanation can often be both frustrating and frightening.

Apart from intrauterine growth restriction/placental insufficiency, infection, malformations and other medical conditions, there were indications that if the clinical routines were better/followed there might have been a chance to prevent the stillbirth in some of the women who were classified as having a possibly or probably preventable stillbirth. According to our assessment, in some of the cases more frequent ultrasound/clinical check-ups, earlier induction of labour and earlier interventions in line with current guidelines were suggested. This is in line with earlier published data that underlined that the quality of care is of great importance before the stillbirth has occurred and that substandard care contributes to 20–30% of stillbirths [13].

According to an analysis of the ANC patient charts, thorough information on the importance of feeling foetal movements and how to act in case of reduced movements was usually given by the responsible midwife. In most cases, this was well documented in the woman’s medical files. A survey conducted by the Swedish National Board of Health and Welfare was sent to the midwives in primary care, and it showed good adherence to the guidelines regarding reduced foetal movements, for example. However, the respondents reported there were factors affecting the implementation of guidelines, such as language barriers [41]. In a previous study of women seeking medical care for reduced foetal movements published by our research group in 2020, [42] it was found that in a very large percentage of stillbirths reduced foetal movement was the symptom of intrauterine death.

Recently, all major obstetrics and gynaecology associations, such as the Royal College of Obstetrics and Gynaecology (RCOG) and the American College of Obstetrics and Gynaecology (ACOG), have highlighted the importance of quality care for patients with stillbirths [43, 44]. In this audit, we could identify elements of substandard care after the stillbirth occurred, such as incomplete diagnostic protocols in 17.7% of the cases. In 20.3% of the cases, an assessment of the cause of stillbirth and planning for the next pregnancy was missing from the patients’ medical files. Being able to plan future pregnancies is one way to provide reassurance to parents [45].

The most common diagnosis associated with stillbirth in this audit was a growth restriction/placenta insufficiency. Following foetal growth during pregnancy is important, and symphysis-fundal height (SFH) is the main metric used in every Swedish ANC unit for identifying SGA foetuses. However, a Cochrane review from 2015 showed there is insufficient evidence that SFH is effective in detecting SGA [46]. Indeed, it has been shown that ultrasound is superior, [47] which is why women in several risk groups (e.g. women with hypertension, women with high BMI) routinely undergo extra ultrasound in addition to what is included in the ANC basic program. However, not all SGA foetuses are identified, which is why better screening routines are needed. Signs of growth restriction seem to be a major risk factor for stillbirth, and ultrasound fetometry could increase the identification of SGA [48]. Even if a large proportion of the stillbirths are SGA, there is evidence that the vast majority of antepartum deaths result from normal size neonates [49, 50]. There are several studies recently published on the identification of subclinical impairment of the placental function in normally grown neonates by evaluating the cerebral placental ratio (CPR) [51–54]. However, identification of normally grown fetuses at risk for adverse antepartum events is still unresolved and these conditions are likely to account for the majority of the unexplained causes of stillbirth.

## Conclusion

This study found that implementing a regional multidisciplinary audit can result in valuable information and can help identify avoidable factors that could contribute to a possible decrease in the rate of stillbirth. This can be done by standardizing the classification, documentation and investigation of stillbirths and by improving the quality of care for these patients. Women with no knowledge of Swedish are at risk of experiencing stillbirth. This study cannot identify the best course of action to help this group, although additional antenatal visits and having good translation services seem to be of importance. We can speculate that a high standard of clinical communication with patients (in the native language of the patient) could, potentially, reduce healthcare related delays, improve attendances and improve parental engagement with the pregnancy.

## Data Availability

In order to protect the people included in the study, our dataset cannot be made available. There are a few patients with very detailed medical information and there is a risk these people could be identified.

## Abbreviations

ANC: Antenatal care
AS: Acute cesarean
BMI: Body mass index
CTG: Cardiotocography
ES: Elective cesarean
IUFD: Intrauterine fetal death
IUGR: Intrauterine Growth Restriction
IVF: In vitro fertilization
RFM: Reduced fetal movements
SFH: Symphysis-fundal height
SGA: Small for gestational age
WHO: World Health Organization

## References

[1] Lawn JE, Blencowe H, Waiswa P (2016). Stillbirths: rates, risk factors, and acceleration towards 2030. Lancet.

[2] Stockholm:Socialstyrelsen. Statistikdatabas för graviditeter, förlossningar och nyfödda april 2018 [updated april 2018]. https://sdb.socialstyrelsen.se/if_mfr_004/val.aspx (accessed 10/01/2020).

[3] World Health Organization (1993). International statistical classification of diseases and related health problems: ICD-10. Instruction manual.

[4] Swedish National Board of Health and Welfare. Cause of death. http://www.socialstyrelsen.se/statistics/statisticaldatabase/help/causeofdeath.

[5] Tveit JV, Saastad E, Stray-Pedersen B (2009). Reduction of late stillbirth with the introduction of fetal movement information and guidelines - a clinical quality improvement. BMC Pregnancy Childbirth.

[6] Lee C (2012). ‘She was a person, she was here’: the experience of late pregnancy loss in Australia. J Reprod Infant Psychol.

[7] Kavanaugh K, Hershberger P (2005). Perinatal loss in low-income African American parents. J Obstet Gynecol Neonatal Nurs.

[8] Gold KJ, Sen A, Hayward RA (2010). Marriage and cohabitation outcomes after pregnancy loss. Pediatrics..

[9] Gold KJ, Kuznia AL, Hayward RA (2008). How physicians cope with stillbirth or neonatal death: a national survey of obstetricians. Obstet Gynecol.

[10] Chan MF, Lou FL, Zang YL (2007). Attitudes of midwives towards perinatal bereavement in Hong Kong. Midwifery..

[11] Mistry H, Heazell AE, Vincent O (2013). A structured review and exploration of the healthcare costs associated with stillbirth and a subsequent pregnancy in England and Wales. BMC Pregnancy Childbirth.

[12] Heazell AEP, Siassakos D, Blencowe H (2016). Stillbirths: economic and psychosocial consequences. Lancet.

[13] Flenady V, Wojcieszek AM, Middleton P (2016). Stillbirths: recall to action in high-income countries. Lancet.

[14] Froen JF, Cacciatore J, McClure EM (2011). Stillbirths: why they matter. Lancet.

[15] Bhutta ZA, Yakoob MY, Lawn JE (2011). Stillbirths: what difference can we make and at what cost?. Lancet.

[16] Pattinson R, Kerber K, Buchmann E (2011). Stillbirths: how can health systems deliver for mothers and babies?. Lancet.

[17] Po G, Monari F, Zanni F (2019). A regional audit system for stillbirth: a way to better understand the phenomenon. BMC Pregnancy Childbirth.

[18] Eskes M, Waelput AJM, Erwich J (2014). Term perinatal mortality audit in the Netherlands 2010–2012: a population-based cohort study. BMJ Open.

[19] Willcox ML, Price J, Scott S, et al. Death audits and reviews for reducing maternal, perinatal and child mortality. Cochrane Database Syst Rev. 2020;3. 10.1002/14651858.CD012982.pub2 PMC7093891 32212268

[20] Norris T, Manktelow BN, Smith LK (2017). Causes and temporal changes in nationally collected stillbirth audit data in high-resource settings. Semin Fetal Neonatal Med.

[21] Swedish National Board of Health and Welfare (2003). The Swedish Medical Birth Register: a summary of content and quality.

[22] Marsal K, Persson PH, Larsen T (1996). Intrauterine growth curves based on ultrasonically estimated foetal weights. Acta Paediatr.

[23] SBU (1998). Routine ultrasound examination during pregnancy.

[24] Ludvigsson JF, Otterblad-Olausson P, Pettersson BU (2009). The Swedish personal identity number: possibilities and pitfalls in healthcare and medical research. Eur J Epidemiol.

[25] Swedish National Board of Health and Welfare. Minskade fosterrörelser 2016. https://www.socialstyrelsen.se/globalassets/sharepoint-dokument/artikelkatalog/kunskapsstod/2016-10-9.pdf (accessed 20/11/2019).

[26] (PPIP) TPPIP. Codes & description: Avoidable factors identified with perinatal death. http://www.kznhealth.gov.za/chrp/documents/Quality%20Improvement/Audit/PPIP/Code%20Lists/PPIP2%20AvFact%20codes.pdf (accessed 10/12/2019).

[27] Varli IH, Petersson K, Bottinga R (2008). The Stockholm classification of stillbirth. Acta Obstet Gynecol Scand.

[28] Robertson L, Knight H, Prosser Snelling E (2017). Each baby counts: national quality improvement programme to reduce intrapartum-related deaths and brain injuries in term babies. Semin Fetal Neonatal Med.

[29] World Health Organisation (2016). Making every baby count: Audit and review of stillbirths and neonatal deaths.

[30] Flenady V, Koopmans L, Middleton P (2011). Major risk factors for stillbirth in high-income countries: a systematic review and meta-analysis. Lancet.

[31] Ekeus C, Cnattingius S, Essen B (2011). Stillbirth among foreign-born women in Sweden. Eur J Pub Health.

[32] Ravelli ACJ, Tromp M, Eskes M (2011). Ethnic differences in stillbirth and early neonatal mortality in the Netherlands. J Epidemiol Community Health.

[33] Penn N, Oteng-Ntim E, Oakley LL (2014). Ethnic variation in stillbirth risk and the role of maternal obesity: analysis of routine data from a London maternity unit. BMC Pregnancy Childbirth.

[34] Drysdale H, Ranasinha S, Kendall A (2012). Ethnicity and the risk of late-pregnancy stillbirth. Med J Aust.

[35] Campbell DA, Lake MF, Falk M (2006). A randomized control trial of continuous support in labor by a lay doula. J Obstet Gynecol Neonatal Nurs.

[36] Gruber KJ, Cupito SH, Dobson CF (2013). Impact of doulas on healthy birth outcomes. J Perinat Educ.

[37] Vonderheid SC, Kishi R, Norr KF, et al. Group prenatal care and doula care for pregnant women. In: Handler A, Kennelly J, Peacock N, editors. Reducing Racial/Ethnic Disparities in Reproductive and Perinatal Outcomes: The Evidence from Population-based Interventions. New York: Spriger publishing; 2011. p. 369–99.

[38] Gardosi J, Kady SM, McGeown P (2005). Classification of stillbirth by relevant condition at death (ReCoDe): population based cohort study. BMJ.

[39] Vergani P, Cozzolino S, Pozzi E (2008). Identifying the causes of stillbirth: A comparison of four classification systems. Am J Obstet Gynecol.

[40] Ellis A, Chebsey C, Storey C (2016). Systematic review to understand and improve care after stillbirth: a review of parents’ and healthcare professionals’ experiences. BMC Pregnancy Childbirth.

[41] Swedish National Board of Health and Welfare (2018). Dödfödda barn-En inventering och förslag på åtgärder.

[42] Sterpu I, Pilo C, Koistinen IS, et al. Risk factors for poor neonatal outcome in pregnancies with decreased fetal movements. Acta Obstet Gynecol Scand. 2020. 10.1111/aogs.13827 32072616

[43] Gynaecologists RCoOa (2010). Green-top guideline 55—late intrauterine fetal death and stillbirth.

[44] Metz TD, Berry RS, Fretts RC (2020). Obstetric care consensus #10: management of stillbirth: (replaces practice bulletin number 102, March 2009). Am J Obstet Gynecol.

[45] Meaney S, Everard CM, Gallagher S (2017). Parents' concerns about future pregnancy after stillbirth: a qualitative study. Health Expect.

[46] Robert Peter J, Ho JJ, Valliapan J (2015). Symphysial fundal height (SFH) measurement in pregnancy for detecting abnormal fetal growth. Cochrane Database Syst Rev.

[47] Harding K, Evans S, Newnham J (1995). Screening for the small fetus: a study of the relative efficacies of ultrasound biometry and symphysiofundal height. Aust N Z J Obstet Gynaecol.

[48] Williams M, Turner S, Butler E (2018). Fetal growth surveillance - current guidelines, practices and challenges. Ultrasound..

[49] Poon LCY, Tan MY, Yerlikaya G, Syngelaki A, Nicolaides KH (2016). Birth weight in live births and stillbirths. Ultrasound Obstet Gynecol.

[50] LCY P, Volpe N, Muto B, Syngelaki A, Nicolaides KH. Birthweight with Gestation and Maternal Characteristics in Live Births and Stillbirths. Fetal Diagn Ther. 2012;32(3):156–65. 10.1159/000338655 22846512

[51] Khalil A, Morales-Rosello J, Townsend R, Morlando M, Papageorghiou A, Bhide A, Thilaganathan B (2016). Value of third-trimester cerebroplacental ratio and uterine artery Doppler indices as predictors of stillbirth and perinatal loss. Ultrasound Obstet Gynecol.

[52] Prior T, Mullins E, Bennett P, Kumar S (2013). Prediction of intrapartum fetal compromise using the cerebroumbilical ratio: a prospective observational study. Am J Obstet Gynecol.

[53] Dall'Asta A, Ghi T, Rizzo G, Cancemi A, Aloisio F, Arduini D, Pedrazzi G, Figueras F, Frusca T (2019). Cerebroplacental ratio assessment in early labor in uncomplicated term pregnancy and prediction of adverse perinatal outcome: prospective multicenter study. Ultrasound Obstet Gynecol.

[54] Kumar S, Figueras F, Ganzevoort W, Turner J, McCowan L (2018). Using cerebroplacental ratio in non-SGA fetuses to predict adverse perinatal outcome: caution is required. Ultrasound Obstet Gynecol.

